# Clinical and radiographic outcomes of horizontal auto-augmentation of the anterior mandible using a single-site surgical technique prior to delayed implant placement: a case series

**DOI:** 10.1186/s12903-026-09134-4

**Published:** 2026-07-11

**Authors:** Hossam Saleh, Riham Eldibany, Dina Metawie

**Affiliations:** Department of oral and maxillofacial surgery, Faculty of Dentistry, Alexandria University, Champlion Street, El-Azarita, Alexandria, Egypt

**Keywords:** Horizontal augmentation, Split bone block, Dehiscence, Symphysis graft

## Abstract

**Background:**

Insufficient bone width is a common obstacle when placing dental implants. Numerous methods have been devised to enhance bone thickness. Autogenous bone is considered the benchmark material owing to its superior properties. The split bone block technique showed favorable results in bone gain, although donor site morbidity and the need for a second surgical site were major concerns. The study aimed to assess bone augmentation in the anterior mandible, using the symphysis as both the donor and recipient site, while the original technique required two surgeries.

**Patients and methods:**

This prospective, single-center, formal consecutive case series study focused on outcomes and involved 11 patients, each having a bone width of less than 4 mm in the anterior mandibular area. Patients underwent a procedure for horizontal ridge augmentation, with implants being placed after a four-month interval. The study assessed the healing of the surgical site, reported complications, and measured pain levels using a visual analogue scale. It evaluated bone gain in width, primary stability, secondary stability of implants, short-term implant success, and augmented bone stability.

**Results:**

The current study revealed postoperative swelling occurred in 45.5% of patients, while hematoma and flap dehiscence were each observed twice. Visual Analog Scale pain scores decreased over two weeks (*P* < 0.001). Mean radiographic bone width increased from (3.27 ± 0.80 mm) preoperatively to (5.88 ± 0.82 mm) at 4 months (*P* < 0.001). Furthermore, mean implant stability quotient scores improved from 72.80 (3.94) to 88.90 (2.10) (*P* < 0.001). The short-term implant success was 90.9%, and the mean linear augmented bone stability was 88.41% ± 6.24% (median: 90.57%), with individual values ranging from a minimum of 74.39% to a maximum of 94.57%.

**Conclusions:**

The findings of this preliminary case series suggest that the auto-augmentation technique from the mandibular symphysis is a technically feasible protocol for horizontal ridge augmentation. By confining the surgery to a single localized window, it achieves adequate bone gain while minimizing donor-site trauma and optimizing patient recovery profiles.

**Trial registration:**

The trial was registered on 12/09/2024 at clinicaltrials.gov (ID: NCT06727591).

## Introduction

Dental implants are gaining recognition as a preferred method for restoring edentulous areas in the mouth, as supported by numerous clinical studies. They provide advantages over other fixed prosthodontic solutions due to their direct integration with the bone and their reduced impact on adjacent structures [1].

Resorption following tooth extraction is almost inevitable. Studies indicate that the alveolar bone loses up to 50% of its width due to remodelling after extraction [2]. A previous meta-analysis reported that 69.7% of non-molar teeth require some form of augmentation to accommodate implants, compared to 45.9% of molar teeth [3]. The remodelling of the alveolar process post-extraction is a progressive physiological process, with the mandible experiencing a three-to-fourfold increase in the severity of resorption compared to the maxilla. This osteolytic activity begins immediately after tooth loss and continues throughout the patients’ lifetime, with the most significant bone loss typically occurring during the first two years [4]. Clinical assessments conducted by Zmyslowska et al. [5] revealed that 60% of cases of mandibular resorption are classified as severe. Additionally, a morphometric analysis of 152 mandibles showed that the predominant resorptive pattern aligns with the Cawood and Howell Class IV (73.7%) [6]. This “knife-edge” ridge morphology is characterised by preserved vertical height but inadequate buccolingual width, presenting a significant anatomical contraindication for standard, prosthetically driven implant placement without prior ridge augmentation [4].

In recent decades, various techniques have been developed to restore the dimensions of edentulous ridges, facilitating implant placement through augmentation. This process utilizes a range of bone grafting materials along with resorbable and non-resorbable membranes to achieve the necessary bone dimensions [7]. Guided bone regeneration (GBR), which employs bone substitutes alongside an outer membrane, has been associated with a complication rate of 16.8% due to issues such as soft tissue dehiscence and membrane exposure [8]. The most frequently encountered complications following lateral bone augmentation include flap dehiscence and minor infections, which often lead to graft loss in numerous cases [9].

In the literature, authors advocate the need for ridge augmentation when the width of the bone is less than 4 mm. The existence of adequate bone dimensions is an essential condition for prosthetic implant placement, as well as for implant success and survival. Autogenous bone grafts are regarded as the benchmark for the augmentation of edentulous ridges before implant placement, as they combine osteoconductive, osteoinductive, and osteogenic properties, in addition to the fact that they don’t elicit an immune response, that’s why regenerative procedures using autogenous bone yield optimal bone density, implant stability, reliable space maintenance during healing, and a 95% success rate [10–12].

Horizontal bone augmentation in implant dentistry employs various techniques to address deficiencies in the alveolar ridge. Key modalities include guided bone regeneration (GBR), which uses barrier membranes and graft materials to facilitate bone regeneration [13]; ridge splitting, which involves dividing the alveolar ridge to create space for implants [14]; distraction osteogenesis, a technique that gradually expands bone through controlled mechanical distraction [15]; block grafting, which entails the transplantation of a bone block from another site; and the split bone block technique (SBB) [16], which involves harvesting and placing a split block of bone [17]. Each method has specific indications and advantages, making careful assessment crucial for achieving successful outcomes.

The SBB technique is a surgical method used for bone augmentation, particularly in cases of alveolar ridge deficiencies. Developed by Khoury F. and Hanser T [17]., this technique creates a gap between native bone and bone plate, hence the name “split bone block,” that is filled with particulate autogenous bone by splitting a block of bone harvested from the patient and fastening it to the ridge with osteosynthesis screws. Because the graft’s mesenchymal cells and cancellous bone integrate with the host bone in response to local stimuli, this method enables significant gains in bone volume and stability [17].

The present study describes a single-incision modification for SBB horizontal ridge augmentation of the anterior mandible, utilizing autogenous bone harvested locally from the defect site to investigate the clinical feasibility and preliminary proof-of-concept of a single-site donor-recipient protocol. By consolidating both bone harvesting and recipient augmentation within a single localized surgical window, this approach aims to manage horizontal deficiencies while minimizing cumulative tissue trauma and avoiding the secondary donor-site morbidity typically associated with multi-site harvesting.

The current study aimed to evaluate the clinical and radiographic aspects of healing at the surgical site, as well as to analyze postoperative complications, short-term implant success, and both primary and secondary implant stability. Additionally, the investigation measured the gain in alveolar bone width in millimeters (MM) and assessed preliminary augmented bone stability following horizontal ridge augmentation in the anterior mandible, using autogenous split bone blocks harvested from the mandibular symphysis.

## Methods

### Participants and recruitment

The present investigation was a prospective, single-center, consecutive case series reported in accordance with the PROCESS guidelines [18]. It involved patients with ASA scores of 1 and 2 [19]. The study took place between May 2024 and May 2025. Participants were recruited from the outpatient clinic of the oral and maxillofacial surgery department at Alexandria University’s Faculty of Dentistry. The objective was to restore missing mandibular anterior teeth in patients with bone widths measuring less than 4 mm. The treatment employed was the split bone block technique, utilizing the chin as a donor site.

### Participants’ eligibility criteria

#### Patients included [20–21]

- Patients with one to two missing anterior mandibular teeth.
- Horizontal bone width < 4 mm.
- Adequate zone of keratinized tissue.

#### Patients excluded [22–23]

- Presence of active or chronic periapical pathoses in adjacent teeth.
- Insufficient inter-arch distance.
- Factors such as bruxism, clenching, alcoholism, and smoking.
- Patients with systemic diseases or medications affecting bone metabolism, including uncontrolled diabetes mellitus, severe osteoporosis, ongoing radiation therapy, and the use of bone-altering medications (e.g., bisphosphonates or long-term corticosteroids).
- Poor oral hygiene, indicated by a plaque index of ≥ 1.
- Active or untreated periodontal disease.

### Sample size

Sample size was calculated assuming a 95% confidence interval, a 5% alpha error, and 80% study power to detect a difference in bone width after horizontal augmentation using a split bone block. Botros et al. [24] reported a mean and standard deviation (SD) pre-augmentation bone width of 3.44 (0.84) and 7.96 (1.73) 4 months post-operatively after ridge augmentation using split bone block. The calculated mean (SD) difference was 4.52 (1.29), and the 95% confidence interval was 3.24, 5.80. Based on a comparison of paired means, the sample size was estimated to be 10 patients, increased to 11 to make up for cases lost to follow-up [12]. Sample size was calculated using MedCalc Statistical Software version 19.0.5 (MedCalc Software bvba, Ostend, Belgium; https://www.medcalc.org; 2019).

### Intervention

The study was conducted on 11 participants who wanted to restore anterior mandibular teeth using implants, and the ridge width was not suitable for prosthetic implant placement. All participants underwent horizontal ridge augmentation at the anterior mandible using the SBB technique, and the donor site was the chin.

### Preintervention patient optimization

#### Preoperative assessment and clinical examination

##### History

A detailed patient history was recorded, including name, age, and gender. All past medical and dental history was recorded.

##### Clinical examination

The clinical evaluation involved both internal and external assessments via:

- Visual Examination: Checking for facial or oral asymmetry, inflammation, occlusal discrepancies, mucosal ulcers, tissue overgrowth, or active fistulas.
- Manual Palpation: Assessing the labial, buccal, and lingual tissues, as well as the augmented site, four months post-procedure, to ensure readiness for implant insertion.
- Baseline oral hygiene was evaluated using the Silness and Löe Plaque Index (PI) [25]. Periodontal health was assessed via probing pocket depths (PPD) and bleeding on probing (BOP). Following the 2017 World Workshop Classification of Periodontal and Peri-Implant Diseases, inclusion required a clinically healthy periodontium, defined as BOP < 10% and no preoperative probing ≥ 4 mm within the surgical quadrant [26].

##### Radiographic examination

A screening orthopantomogram for each patient to exclude any bone pathology and help in case selection, followed by cone beam computed tomography (CBCT) using I-CAT Next generation imaging device (I-Cat, Imaging Sciences International, Hatfield, Pennsylvania) to evaluate the donor site, all the important vital structures e.g.: mental foramen and lingual canal, also for evaluation of bone dimensions before augmentation procedure then 4 months after augmentation and 3 months following implant placement.

### Intervention and intervention specifics

#### Preoperative patient preparation

Supra- and sub-gingival instrumentation was performed as needed, 7 days prior to the surgery. Patients were instructed to use an antiseptic mouthwash containing chlorhexidine for 1 min before the procedure (Chlorhexidine 125 mg/100 ml, concentration 0.125%).

#### Operative procedure

**Fig. 1:**
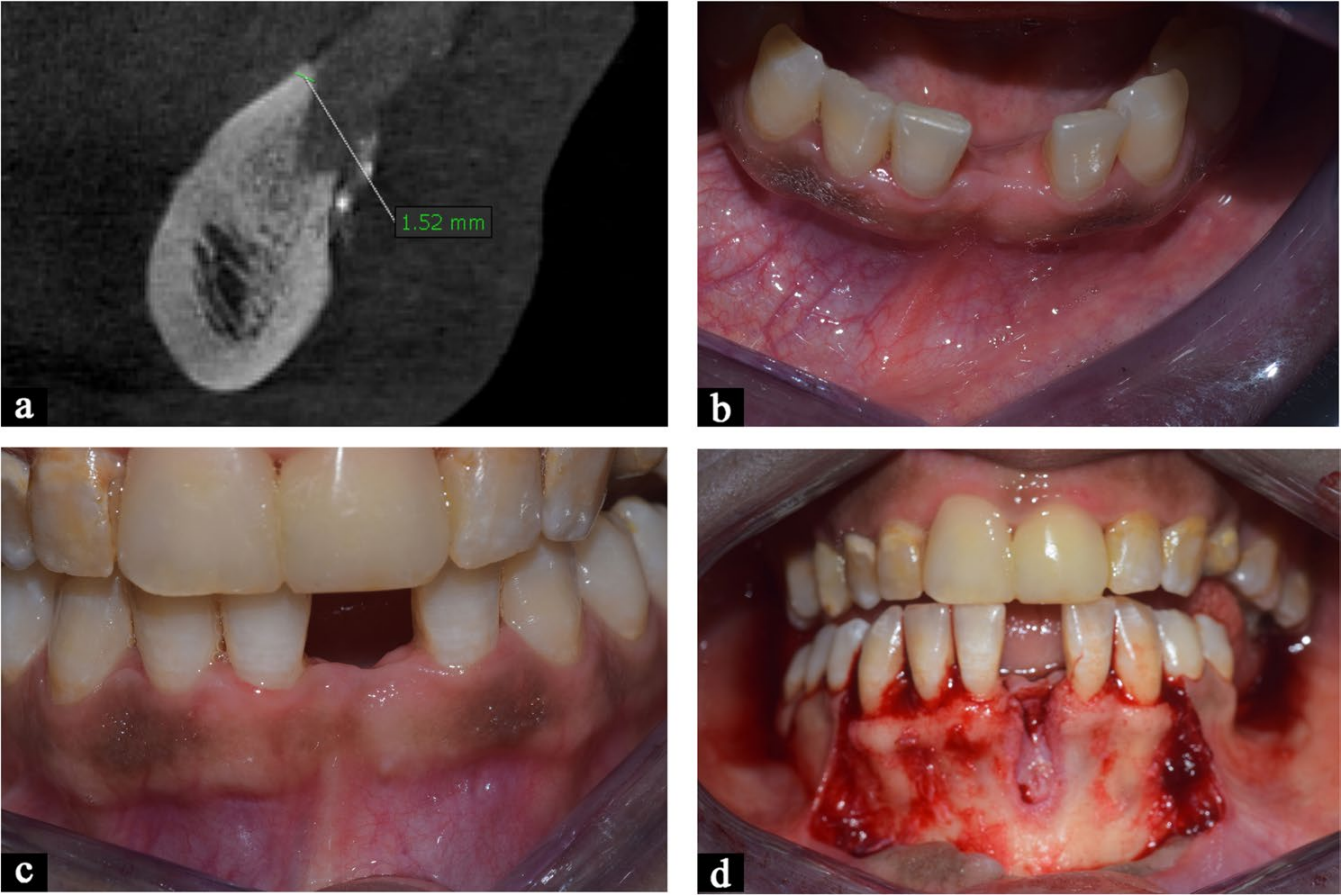
**a** Preoperative CBCT from DVR module; note radiopaque artefact from previous failed root canal treatment **b** Preoperative situation occlusal view **c** Preoperative situation frontal view **d** Flap elevation

All surgeries were done under local anesthesia (Articaine 4% with epinephrine 1:100000, ArtPharma Pharmaceuticals, Egypt). Double mental nerve block and lingual infiltration were used to properly anesthetize both the donor and recipient sites. Blade no. 15c with a Bard-Parker No. 3 Scalpel handle was used to perform a crestal, intrasulcular incision and two vertical incisions. Generally, the incision extended one or more teeth distal to the surgical site and 5 mm away from the mental foramen. Full thickness mucoperiosteum flap was elevated using a periosteal elevator. Fig. 1. 

**Fig. 2:**
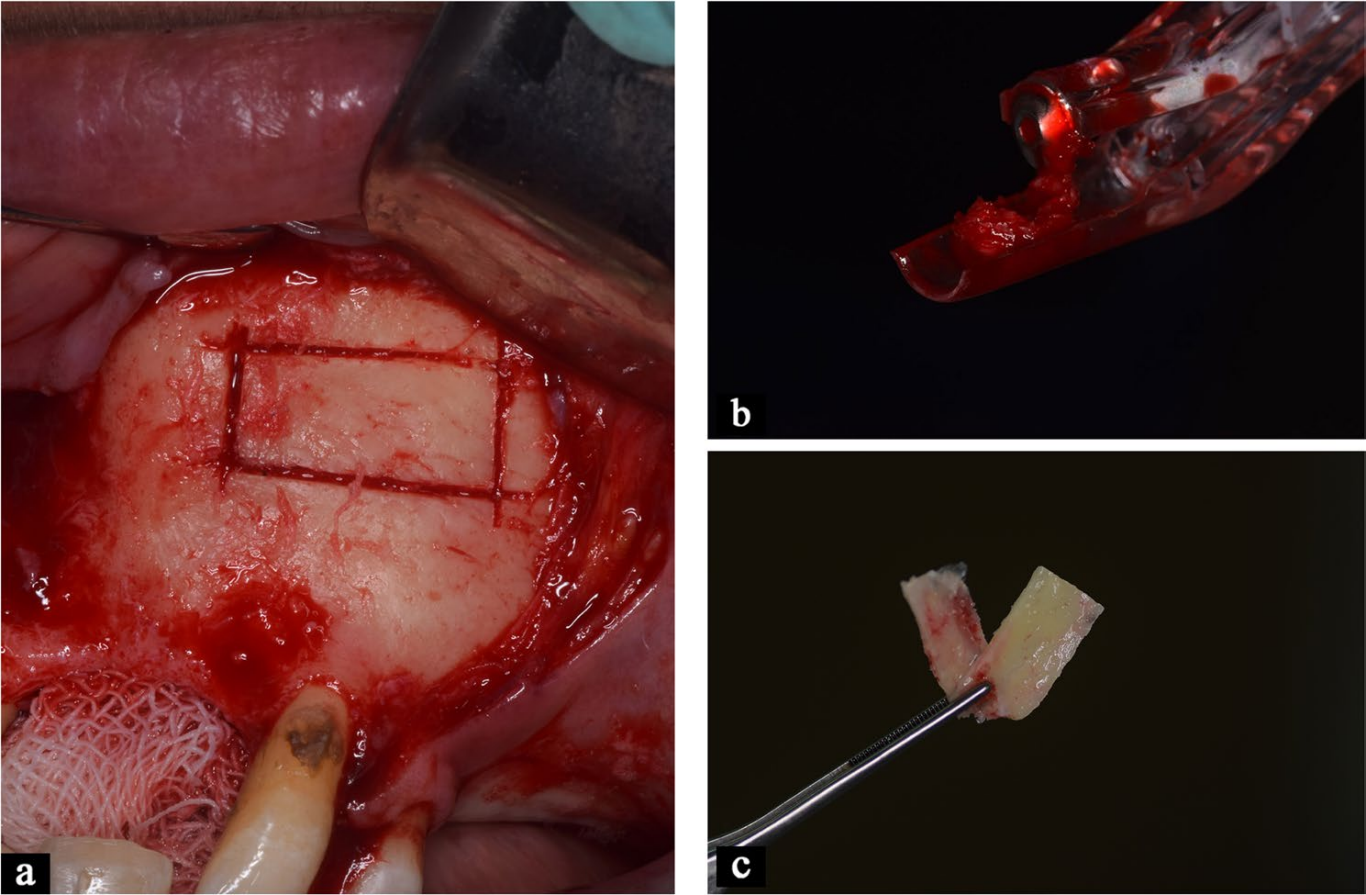
**a** Unicortical cuts made by piezotome for graft harvesting. **b** Bone block split into two bone plates **c** Bone scraper harvesting particulate autogenous bone

Intraoperatively, the patterning of the defect was done to accurately determine the dimensions of the bone to be harvested from the donor site. At the symphysis area, unicortical cuts were made. The cuts were made using the bone surgery tip supplied by the Piezotome Cube Surgery Unit (Acteon group, Merignac, France). The BS1S sharp saw tip, which is 9 mm long, was used to create a unicortical cut in the bone. The cuts were made at least 5 mm inferior to root tips, 5 mm superior to the inferior border of the mandible, and 5 mm away from the mental foramen. Unibeveled bone chisel (Devemed GmbH, Neuhausen obeck, Germany) was used to luxate the graft. The donor site and the graft were scraped for particulate bone chips using Easy Bone Scrapper (Dental Studio Co Ltd, Gyeonggi-do, Republic of Korea). Decortication of the recipient site was done using a size two surgical bur or a round rose head bur. The graft was split into two bone plates 1–2 mm using a diamond disc under a copious amount of irrigation. Fig. 2. 

**Fig. 3:**
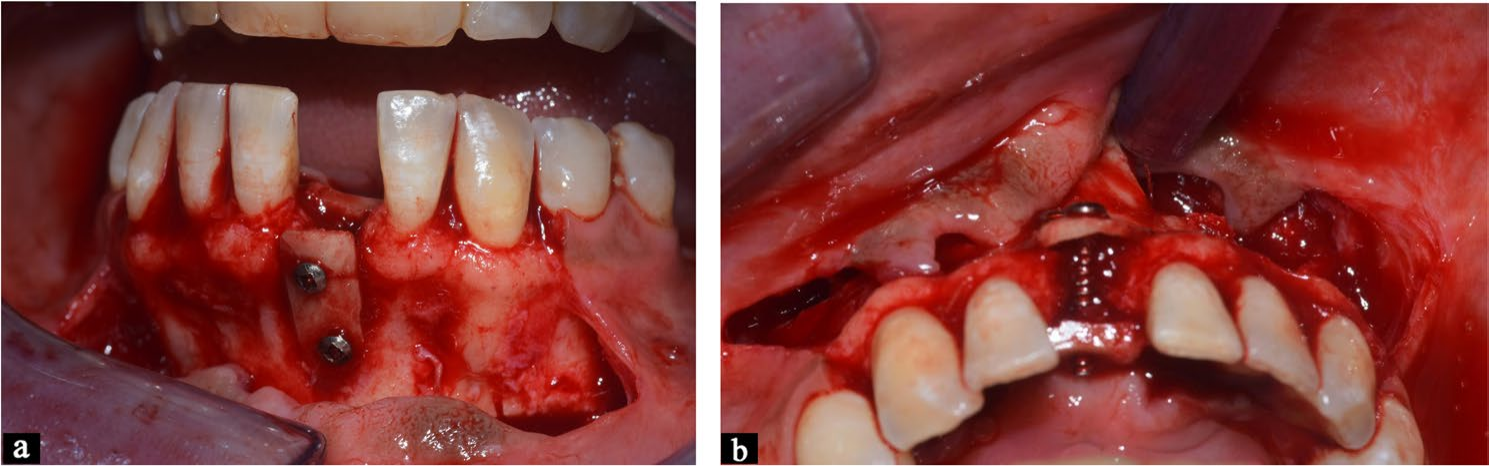
**a** Stabilization of the bone plate frontal view **b** Stabilization of the bone plates occlusal view

The bone plates were stabilized into the recipient site using 2 titanium screws (Biomaterials Korea, Seoul, Republic of Korea SK V1 Center 402) placed midway coronally-apically and/or mesial-distally. The screws’ diameter used ranged from 1.3 to 1.5 mm. The length of the screw was determined according to the defect being grafted. One bone plate was placed labially in mild horizontal ridge defect cases, whereas two bone plates were placed labially and lingually in moderate-to-severe horizontal ridge defect cases to aid in prosthetically driven implant placement Fig. 3.

**Fig. 4:**
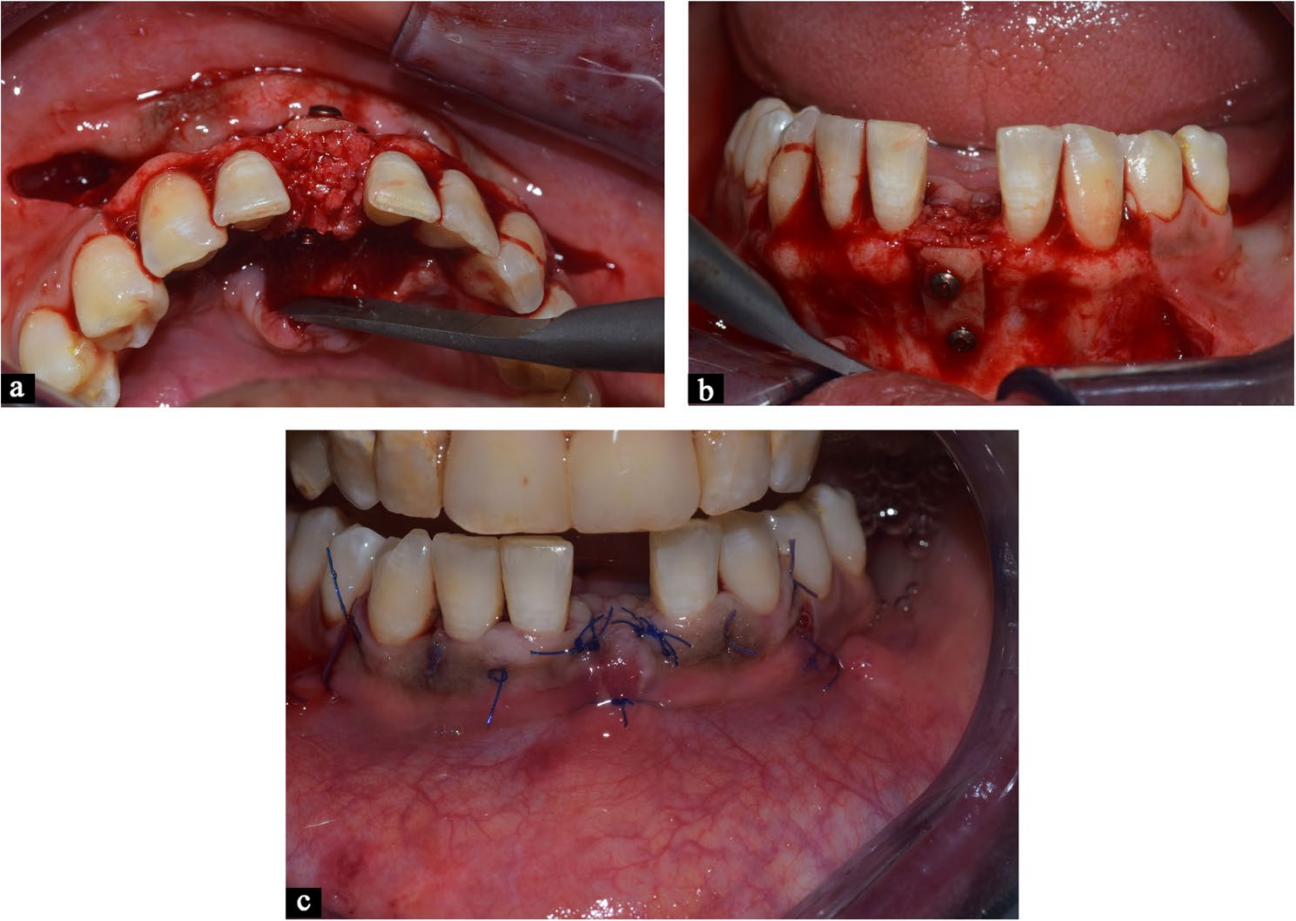
**a** Particulate bone packed between bone plates, frontal view **b** Particulate bone packed between bone plates, occlusal view **c** Flap closure

The autogenous bone particles were packed into the space created by the bone plates. A modified periosteal releasing incision was performed using blade no. 15c, not more than 1 mm in depth, near the apical part of the flap; then, lateral stretching using the convex smooth side of the periosteal elevator was used to aid in tension-free flap closure. A collagen sponge was packed into the donor site. The flap was sutured using 4 × 0 or 5 × 0 non-resorbable synthetic suture material. Tension-free closure was achievable using 1 apical horizontal mattress and two simple interrupted sutures at the edentulous space. Simple interrupted sutures were used at the two vertical incisions, and vertical mattress sutures were used between teeth [27] Fig. 4.

**Fig. 5:**
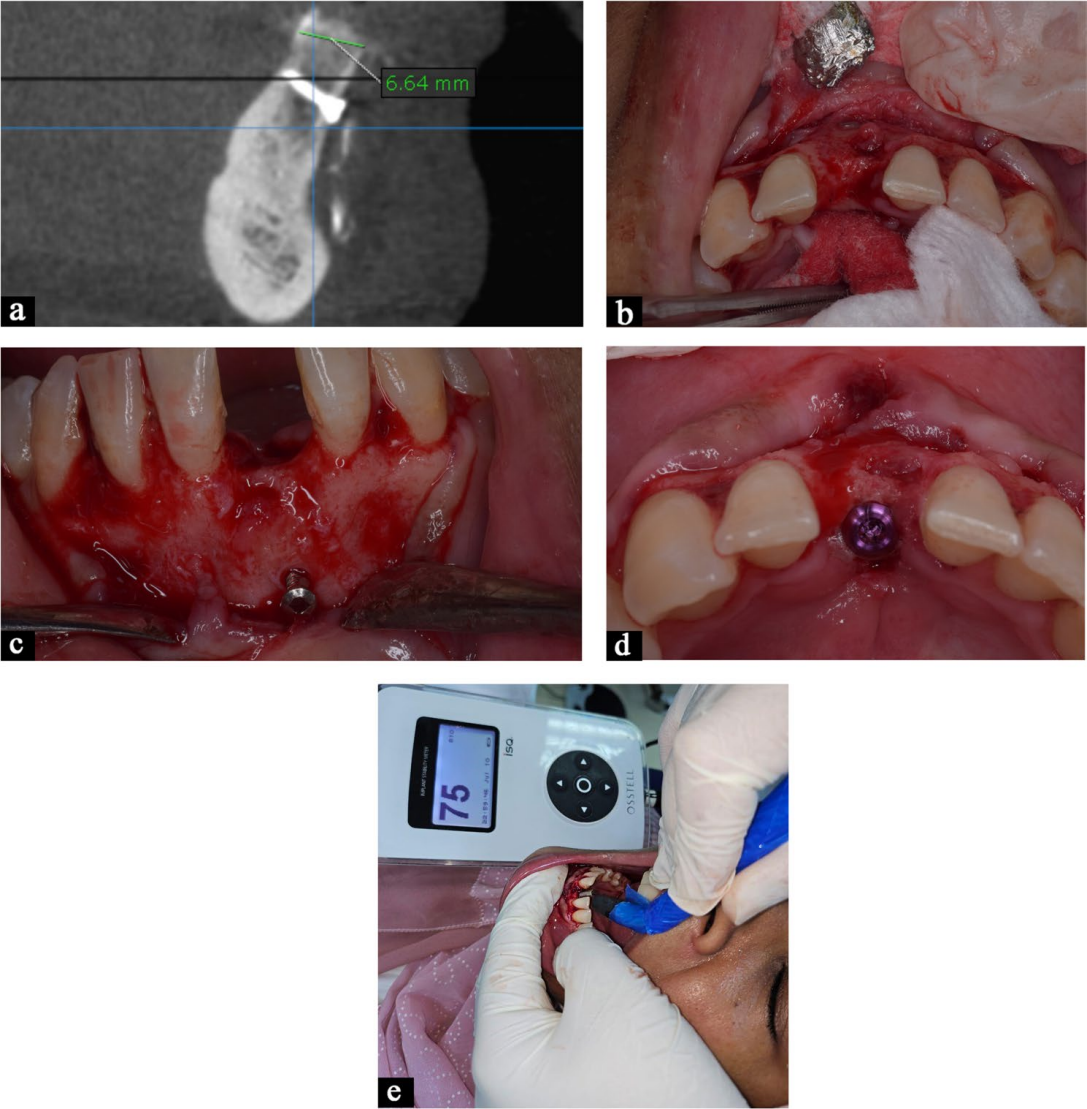
**a** Postperative CBCT from DVR module **b** Flap design for implant placement occlusal view **c** Flap design for implant placement frontal view **d** Implant placement **e** Osstell reading for primary stability

Four months later, the site was accessed using a crestal incision for implant placement. Implant (Vitronex dental Implant VIA F. Turati, Milano, Italy) was placed according to the prosthetic plan. The implant was placed 1 mm subcrestal. The implants were placed at 35Ncm torque according to the manufacturer’s guidelines. All implants placed had the same diameter, 3.5 mm. Primary stability was measured using Osstell ISQ (Osstell, Gothenburg, Sweden) Fig. 5.

**Fig. 6:**
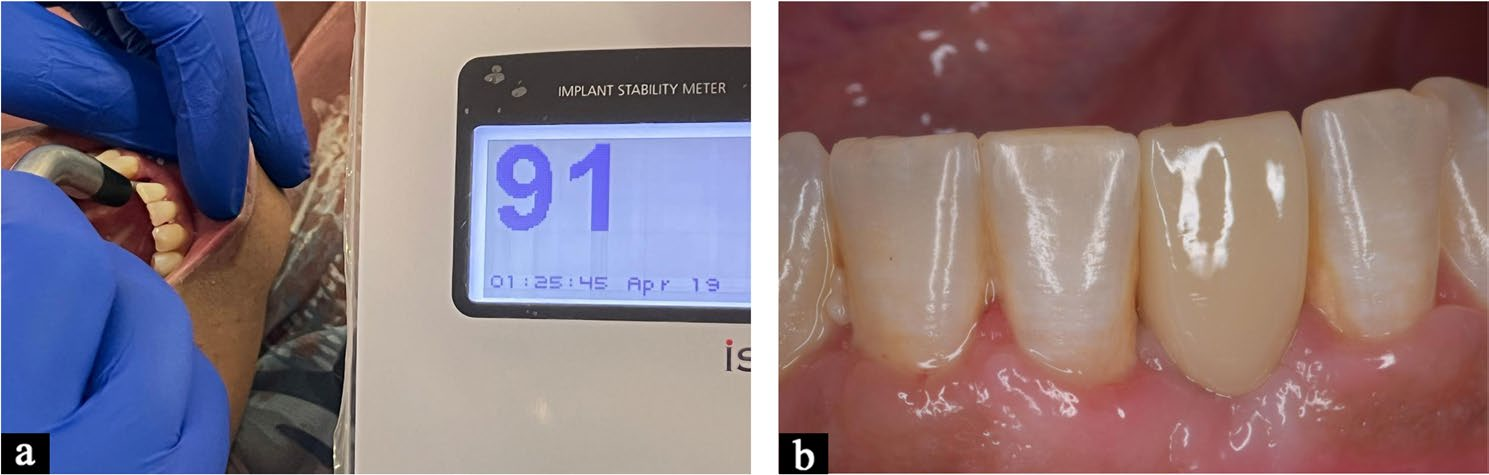
**a** Secondary stability **b** Crown delivery

Implants were left for 3 months for healing. After 3 months, the implants were uncovered using a crestal incision. Secondary stability was measured. Healing abutments were placed for 14 days. A conventional open tray impression was made, and a zirconia crown/bridge was delivered Fig. 6. 

### Operator’s details and quality control

To ensure consistency between all the cases. All the operations were performed by a single experienced operator who conducted the study. In our research, CBCT measurements were performed by 2 experienced examiners. For assessment of intra- and inter-examiner reliability, radiographic measurements were independently performed by two examiners under standardized conditions. Intra-examiner reliability was evaluated through repeated measurements after a two-week interval, and ICC analysis demonstrated good to excellent agreement (ICC range: 0.86–0.92).

Additionally, all measurements were performed using standardized imaging protocols and software tools to reduce potential errors.

### Post-operative care and follow-up

#### Postoperative phase

Patients were instructed to apply cold fomentation for the first 24 h, warm saline mouthwash for the second 24 h, enforce good oral hygiene measures, and were regularly followed up for the first 1 month. Sutures were removed after 14 days. The following medications were prescribed.

- Amoxicillin 875 mg + Clavulanic acid 125 mg. 1000 mg every 12 h for 5 days.
- Metronidazole 500 mg every 8 h for 5 days.
- Diclofenac potassium 50 mg every 8 h for 5 days.
- Chymotrypsin 300 EAU – trypsin 300 EAU every 8 h for 5 days.

#### Clinical and radiographic follow-up

A thorough Follow-up was performed for the assessment of the following clinical parameters:

#### Postoperative pain

It was assessed through a 10-point Visual Analogue Scale (VAS) [28]. The patients were asked how they could quantify the pain when 10 was the maximum pain felt and 0 was no pain at all. Pain was measured one week and two weeks after surgery.

#### Postoperative complications

Postoperative complications encountered during the two surgeries, such as oedema, flap dehiscence, or implant failure, were observed, analyzed, and recorded.

### Implant success/survival

Due to the limited follow-up period inherent to the present study, implant outcomes were evaluated in terms of short-term survival and success. Assessment was performed until the last available follow-up visit according to the health scale for dental implants proposed by Carl E. Misch et al. [29], consistent with the Pisa Consensus criteria for implant health and disease. Implant survival was defined as the presence of the implant in situ at the time of follow-up, regardless of biological or technical complications, whereas implant success incorporated both clinical and radiographic parameters of functional implant health. Based on these criteria, implants were categorized into four groups: (A) Success, representing optimal clinical and radiographic conditions; (B) Satisfactory survival, indicating stable function with minor deviations from ideal conditions; (C) Compromised survival, implants with biological and prosthetic complications that remained functional but required monitoring or intervention; and (D) Failure, defined as implants requiring removal or presenting with loss of function.

### Implant stability [30]

Primary implant stability was assessed at day 0 of implant placement for all implants, and secondary implant stability was assessed after 3 months from implant placement for successful and surviving implants, 10 implants, using Osstell dx.

A SmartPeg transducer was attached to the implant fixture to enable resonance frequency analysis (RFA). The Osstell device was utilized to measure the Implant Stability Quotient (ISQ) in both the mesiodistal and buccolingual orientations. In accordance with the manufacturer’s protocol, measurements were deemed stable and recorded only when two identical consecutive values were obtained or when a specific value appeared twice within three consecutive attempts. For statistical analysis, the final ISQ for each implant was calculated as the arithmetic mean of the recorded values across all orientations [31].

Osstell evaluates RFA, which is a measure of implant stability. It has a scale from 1 to 100 where:

- < 60 was considered low stability.
- 60 ~ 69 was considered intermediate stability.
- ≥ 70 was considered high stability.

### Radiographic evaluation

A follow-up CBCT 4 months later using I-CAT Next-generation imaging device (I-Cat, Imaging Sciences International, Hatfield, Pennsylvania) was used for all patients using the same machine under the same exposure parameters: 120 kVp, 5 mA, 26.9 s at 0.25 resolution. CBCT following ridge augmentation for postoperative radiographic evaluation of horizontal bone gain in mm and selection of implant size and location was performed. The output data of the imaging device, the Digital Imaging and Communications in Medicine file (DICOM), was saved. It was processed using On-Demand 3D software. Preoperative CBCT scans were initially evaluated to establish baseline bone width (T1) and stratify the type of bone defect according to Wang HL and Al-Shammari K. HVC ridge deficiency classification [21]. Alveolar ridge defects were categorized using the HVC classification system, which evaluates deficiency based on the pattern of bone loss—Horizontal (H), Vertical (V), or Combined (C). Defects were further classified by severity as mild (≤ 3 mm), moderate (4–6 mm), or severe (≥ 7 mm) to standardize preoperative baselines. This classification is therapeutically oriented and helps guide the selection of an appropriate ridge augmentation procedure and implant planning.

To assess postoperative changes in bone width, a secondary CBCT scan was performed four months after the augmentation procedure (T2). A tertiary CBCT scan (T3) was subsequently acquired three months later to evaluate preliminary stability of the augmented bone.

The CBCT dataset was reoriented so that the axial plane was parallel to the occlusal plane, while the coronal plane was aligned with the long axis of the distal tooth. In the Multiplanar Reformation (MPR) module, the coronal plane was also aligned parallel to the long axis of the tooth located distal to the edentulous space.

**Fig. 7:**
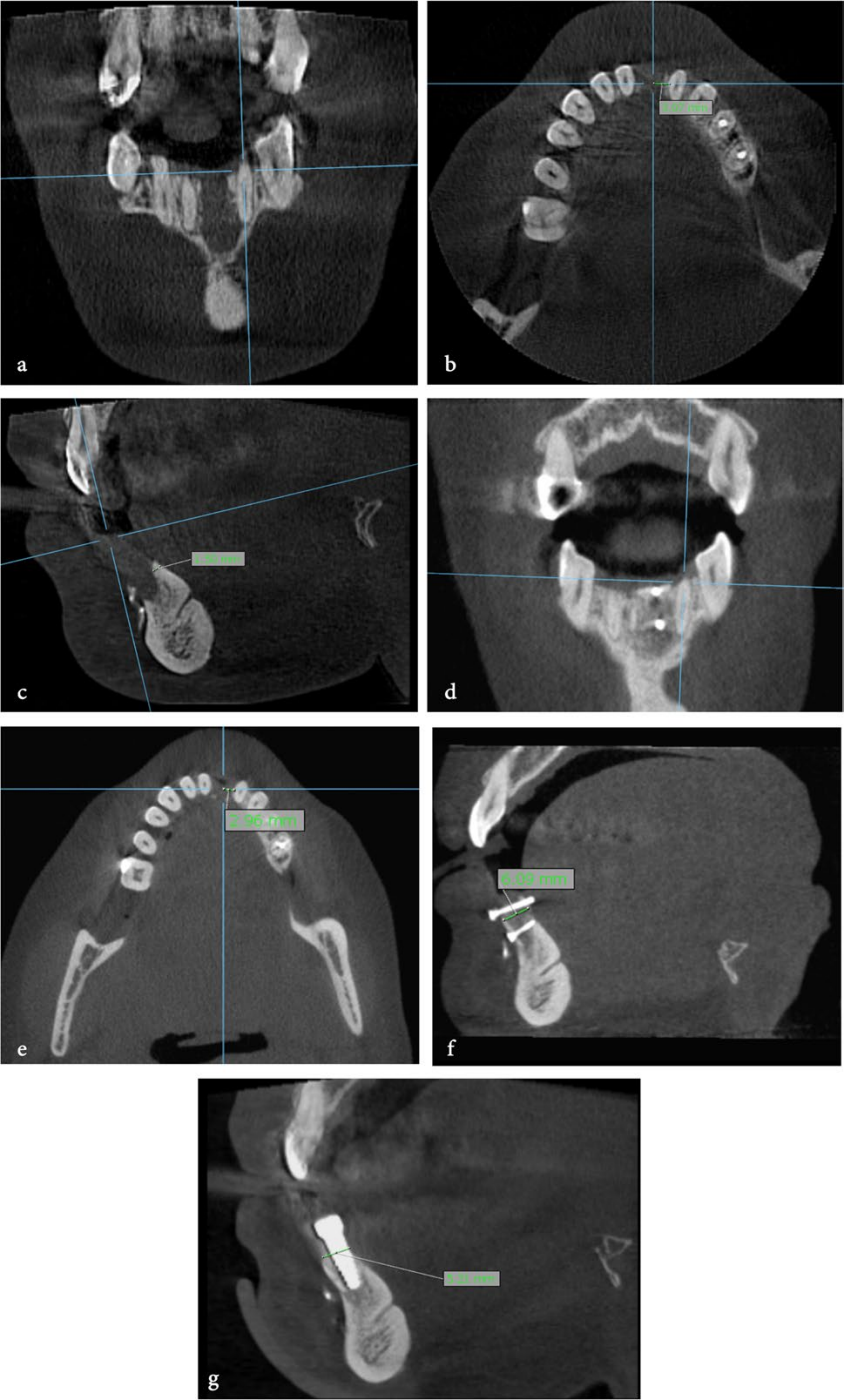
**a** On the MPR module the coronal plane is parallel to long axis of reference tooth to measure preoperative bone width **b** 3 mm distance from reference tooth on cross sectional plane **c** Measurement of T1 **d** On the MPR module the coronal plane is parallel to the long axis of reference tooth to measure postoperative bone width **e** 3 mm distance from reference tooth on cross sectional plane **f** Measurement of T2 **g** Measurement of T3

On the axial view, a point approximately 3 mm from the specified tooth was selected. The bone width was measured on the sagittal view, located 1 mm below the highest point of the bone. To evaluate intra- and inter-examiner reliability, radiographic measurements were conducted independently by two examiners under standardized conditions. Intra-examiner reliability was assessed through repeated measurements taken after a two-week interval, and ICC analysis indicated good to excellent agreement (ICC range: 0.86–0.92) Fig. 7. 

### Follow-up policy

The patients were followed for a minimum of 7 months in the outpatient clinic of the Oral and Maxillofacial Surgery Department at the Faculty of Dentistry. Personal information for all patients was recorded, and direct contact was established with the patients and their relatives through telephone numbers. This approach was taken to facilitate follow-up and ensure that it was thorough and current.

### Statistical analysis

Normality of quantitative variables was assessed using descriptive statistics, Q-Q plots, histograms, and the Shapiro–Wilk test. All data showed a normal distribution, so parametric analysis was adopted. Descriptive statistics were calculated as means, standard deviations (SD), and ranges (Min–Max) for quantitative variables and frequencies and percentages for qualitative variables. Comparisons between pre- (T1) and post-operative (T2) measurements were performed using a paired samples t-test, while comparisons between T1-T3 were performed using repeated measures ANOVA, followed by multiple pairwise comparisons using Bonferroni correction. All enrolled participants completed the study follow-up, and no missing data were encountered during data collection or analysis. Statistical significance was set at a p-value < 0.05. Data were analyzed using IBM SPSS for Windows (Version 26.0).

## Results

### Demographics and baseline clinical status

The study comprised 11 patients, consisting of 7 females and 4 males, with an age range spanning 33 to 64 years. All recruited participants were classified as ASA I or II. Preoperative clinical evaluation confirmed satisfactory oral hygiene and stable periodontal health throughout the entire study. The mean baseline Plaque Index according to Silness and Löe was 0.42 ± 0.15, denoting adequate plaque control. All subjects demonstrated healthy periodontal status before surgical intervention, characterized by full-mouth bleeding on probing (BOP) scores below 10% and localized probing pocket depths (PPD) measuring ≤ 3 mm within the treated segments. The study included 7 patients with mild horizontal bone defects, 3 patients with moderate to severe horizontal bone defects, and 1 patient with a combined defect. All patients underwent a standardized, prosthetically driven workflow utilizing identical implant dimensions driven by the anatomical constraints of the anterior mandible. A comprehensive, case-by-case presentation of individual subject parameters and clinical timelines is detailed in Table 1.

**Table 1:** Comprehensive summary table

Patient	Gender	AGE	Preop HVC Defect	Number of bone plate(s)	Preop Plaque Index	Prop periodontal status	T1	T2	T3	Prosthetic Plan	Complications	Implant status	Primary stability	Secondary Stability	Augmented Bone Stability
1	FEMALE	45	Mild H	1 labial cortical plate	0.3	Healthy BOP ≤ 10%	3.93	5.17	4.65	Screw retained crown	None	Group I	72	91	89.94%
2	MALE	52	Mild H	1 labial cortical plate	0.4	Healthy BOP ≤ 10%	3.92	5.11	4.7	Screw retained crown	Mild swelling resolved in 5 days	Group I	72	90	91.97%
3	FEMALE	38	Mild H	1 labial cortical plate	0.4	Healthy BOP ≤ 10%	3.91	6.53	5.58	Screw retained crown	None	Group I	71	88	85.45%
4	FEMALE	36	C(minor)	2 cortical plates (labial and Lingual)	0.4	Healthy BOP ≤ 10%	1.5	6.64	4.94	Screw retained crown	Fontana Class II CHX mouthwash	Group II	75	90	74.39%
5	MALE	33	Mild H	2 cortical plates (labial and Lingual)	0.6	Healthy BOP ≤ 10%	3.49	7.57		Screw retained crown	hematoma Fontana Class I Lost implant stability	Group IV	48	Failure	Failure
6	FEMALE	48	Mild H	1 labial cortical plate	0.3	Healthy BOP ≤ 10%	3.77	5.26	4.354	Screw retained crown	Mild swelling resolved in 6 days	Group I	77	85	82.77%
7	FEMALE	55	Mild H	1 labial cortical plate	0.5	Healthy BOP ≤ 10%	3.58	5.67	4.93	Screw retained crown	None	Group I	72	87	86.94%
8	MALE	42	Moderate H	2 cortical plates (labial and Lingual)	0.4	Healthy BOP ≤ 10%	2.71	5.9	5.5	Screw retained crown	None	Group II	79	92	93.22%
9	FEMALE	64	Moderate H	2 cortical plates (labial and Lingual)	0.3	Healthy BOP ≤ 10%	2.38	5.12	4.67	Screw retained crown	Hematoma & Mild swelling65	Group I	75	88	91.21%
10	MALE	50	Mild H	1 labial cortical plate	0.5	Healthy BOP ≤ 10%	3.89	6.5	6.09	Screw retained crown	Mild swelling	Group I	65	90	93.69%
11	FEMALE	59	Moderate H	1 labial cortical plate	0.4	Healthy BOP ≤ 10%	2.91	5.3	5.01	Screw retained crown	Mild swelling	Group I	70	88	94.52%

### Postoperative complications

Postoperative complications were mild, localized, and manageable. Mild swelling confined to the single operative zone was the most frequent observation, occurring in 5 out of 11 patients (45.5%) and resolving spontaneously within 7 days. Localized hematoma and minor soft-tissue dehiscence each affected 2 out of 11 patients (18.2%). The reported adverse events and their absolute distribution are summarized in Table 2.

**Table 2:** Post-operative complications

	*N* (%)
Swelling	5 (45.5%)
Hematoma	2 (18.2%)
Dehiscence	2 (18.2%)

Patient-reported pain tracked via the Visual Analog Scale (VAS) demonstrated a favorable downward descriptive pattern over the early healing phase. The mean VAS score decreased from (5.73 ± 1.56) at 1 week postoperatively to (2.18 ± 2.40) at 2 weeks, representing a mean descriptive reduction of 61.95% over the observational period as shown in Table 3.

**Table 3:** Post-operative pain up to two weeks

	Mean (SD)	Min - Max
VAS at 1 week	5.73 (1.56)	3.00–8.00
VAS at 2 weeks	2.18 (2.40)	0.00–7.00
Mean (SD) difference	-3.55 (2.07)	
95% CI	-4.93, -2.16	
% reduction	-61.95%	
*P* value	**< ** **0.001** *	

Paired samples t-test was used

*SD* Standard Deviation, *Min* Minimum, *Max* Maximum, *CI* Confidence Interval

*Statistically significant at *p*-value < 0.05

### Radiographic evaluation

CBCT scans demonstrated an exploratory assessment of horizontal ridge enhancement. The preoperative mean horizontal bone width (T1) was (3.27 ± 0.80 mm). At 4 months post-augmentation (T2), the mean width increased to (5.88 ± 0.82 mm), representing a 79.82% gain in ridge dimension. Following implant placement and intermediate tracking (T3), the mean horizontal bone width was recorded at (5.10 ± 0.55 mm), reflecting a net descriptive width increase of 55.96% from the baseline preoperative width as shown in Table 4.

**Table 4:** Bone width (mm) on CBCT pre-operative and 4 months post-operative

	Mean (SD)	Min - Max
Pre-operative bone width (T1)	3.27 (0.80) **a**	1.50–3.93
Post-operative bone width (T2)	5.88 (0.82) **b**	5.11–7.57
Post-operative bone width (T3)	5.10 (0.55) **c**	4.35–6.08
Mean (SD) difference (T3-T1)	1.81 (1.05)	
95% CI	1.06, 2.56	
% increase	55.96%	
*P* value	**< ** **0.001** *	

Repeated measures ANOVA was used

*SD* Standard Deviation, *Min* Minimum, *Max* Maximum, *CI* Confidence Interval

a, b,c: different letters denote significant differences between timepoints using Bonferroni correction

*Statistically significant at p-value < 0.05

### Implant stability

Resonance Frequency Analysis (RFA) demonstrated a positive clinical outcome between early placement phases. The primary stability mean for the surviving implants was 72.80 ± 3.94 ISQ, while the secondary stability mean increased to 88.90 ± 2.10 ISQ, representing a descriptive increase of 22.12% Table 5.

**Table 5:** Primary and secondary implant stability

	Mean (SD)	Min – Max
Primary stability	72.80 (3.94)	65.00–79.00
Secondary stability	88.90 (2.10)	85.00–92.00
Mean (SD) difference	16.10 (4.51)	
95% CI	12.88, 19.33	
% increase	22.12%	
*P* value	**< ** **0.001** *	

Paired samples t-test was used

*SD* Standard Deviation, *Min* Minimum, *Max* Maximum, *CI* Confidence Interval

*Statistically significant at *p*-value < 0.05

### Implant success/survival

Implant health was indexed using Misch et al. Pisa Consensus Scale. Eight implants (72.7%) achieved complete success (Group I), and two (18.2%) demonstrated satisfactory survival (Group II). One implant failure (9.1%) occurred during early healing due to lost mechanical stability (Group IV).

### Linear augmented bone stability

Complete grafted bone stability data were obtained and analyzed as the percentage of bone remaining at T3 relative to T2 (T3/T2 × 100), demonstrating favorable results. The mean linear augmented bone stability was 88.41% ± 6.24% (median: 90.57%), with individual values ranging from a minimum of 74.39% to a maximum of 94.57%. The results provide early evidence of good maintenance and stability of the augmented bone within the studied cases.

### Intervention adherence and compliance

To ensure patient compliance with instructions, VAS was assessed once weekly for two weeks, emphasizing that all the post-operative instructions and medication were taken on time.

### Complications and adverse events

**Fig. 8:**
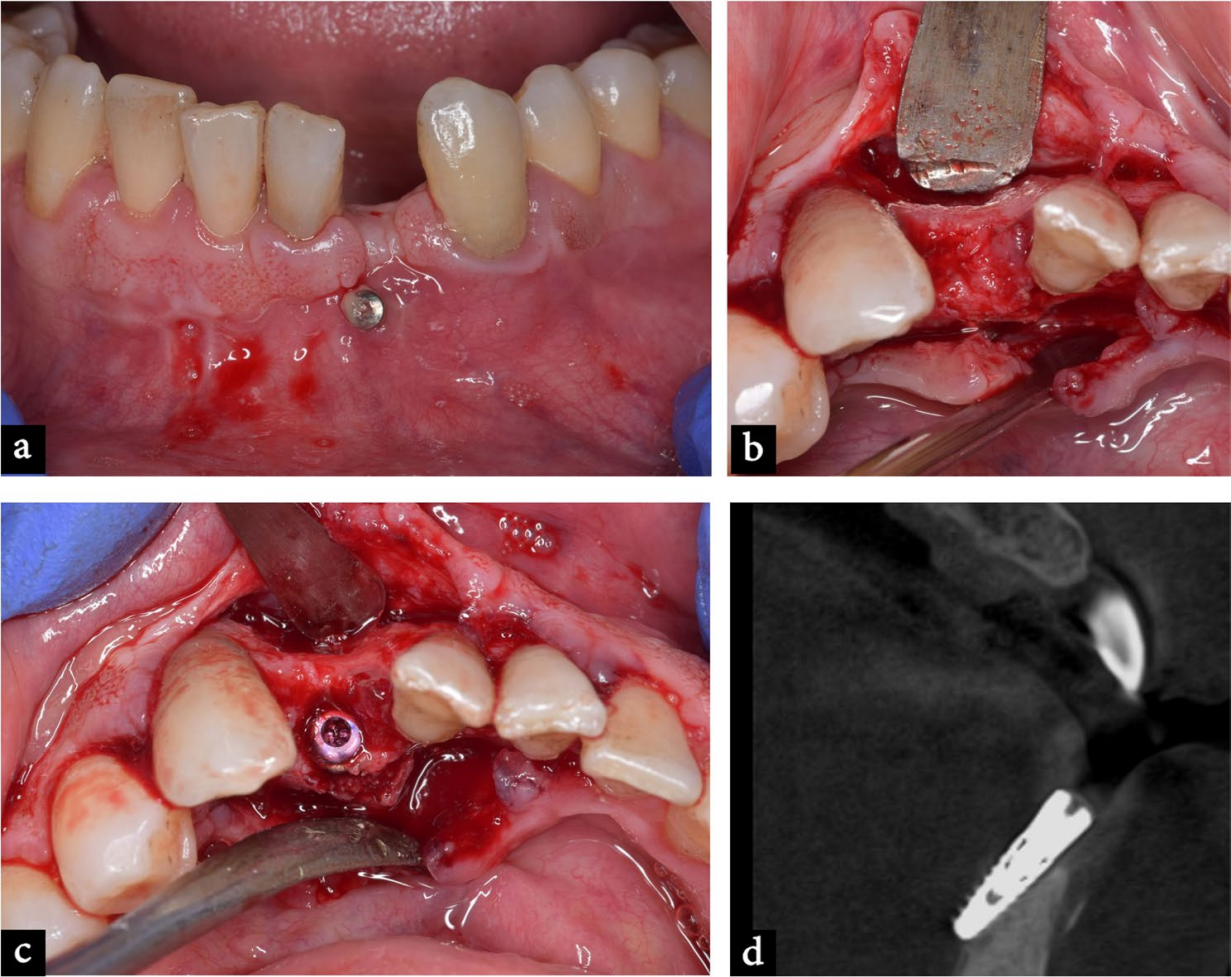
**a** Screw head exposure, Fontana class I dehiscence **b** Flap reflection for implant placement **c** Implant placement and fracture of the lingual plate **d** Postoperative CBCT showing resorption of the lingual plate of bone

Screw head exposure was recorded in one patient. Although the augmentation achieved the intended ridge volume, since the case showed the intended horizontal bone gain, the wound dehiscence may have adversely affected bone maturation and cortical integrity. During the implant placement the lingual cortical plate of bone fractured, and the implant showed a low stability (48), the implant was left for submerged healing, though no infection was noticed during the healing phase. A subsequent CBCT evaluation, conducted three months after implant placement, revealed that the lingual position of the implant fixture had resulted in resorption of the lingual plate. The implant failed at the time of prosthetic loading. Thus, while the augmentation was volumetrically successful, implant placement was classified as a clinical failure Fig. 8. 

**Fig. 9:**
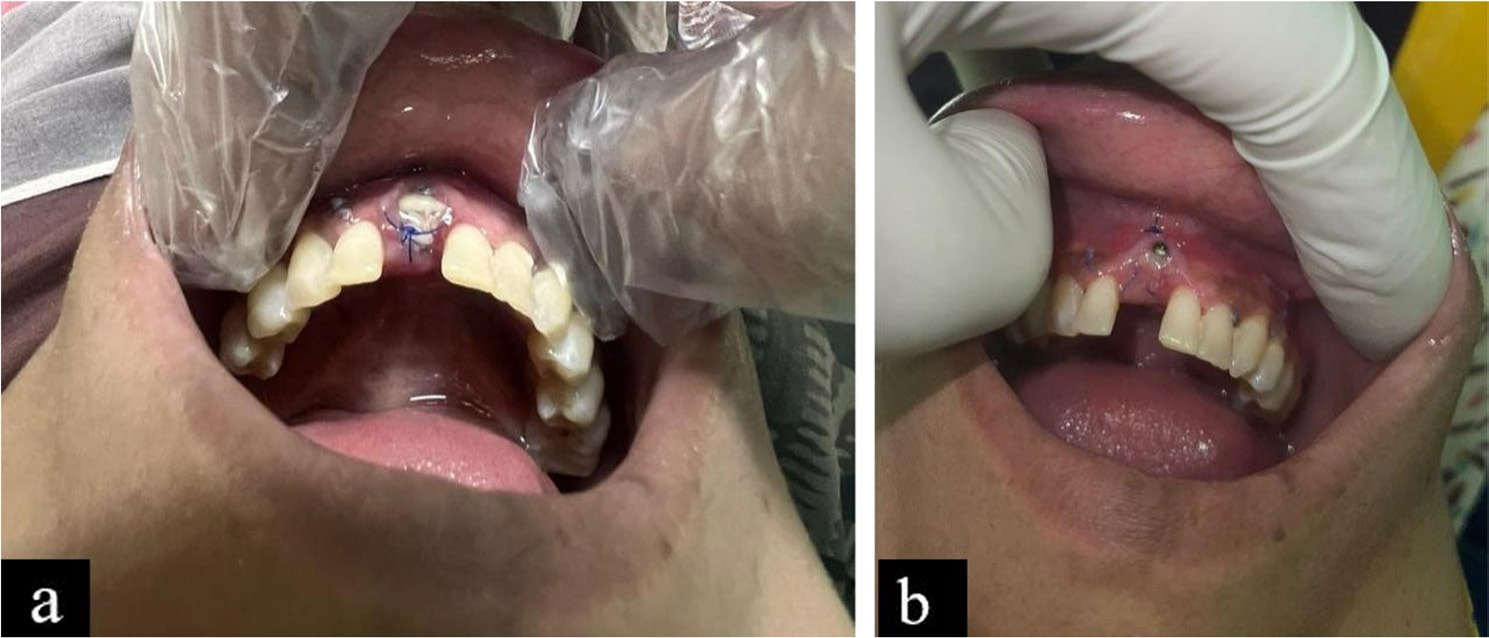
**a** Flap dehiscence **b** Re-epithelialization

Another patient, 14 days postoperatively, presented with flap dehiscence. The site was managed via debridement and mechanical refreshing of the wound edges. Following a one-week regimen of meticulous oral hygiene and topical hyaluronic acid gel application, complete re-epithelialization was observed. The surgical outcome remained successful, with no adverse effects on bone height or density, allowing for implant placement to proceed according to the original timeline Fig. 9.

## Discussion

In 1988, Cawood and Howell [6] conducted an evaluation of 300 dried skulls to characterize the anatomy and morphology of alveolar bone resorption. Their findings indicated that while the basal bone remains relatively stable, the alveolar process experiences significant resorption. By measuring the horizontal and vertical components at specific anatomical landmarks (points S, K, and M), they classified ridges with sufficient vertical height but insufficient horizontal width as Class IV defects [6]. In a subsequent study involving 152 mandibles, the authors concluded that mandibular resorption predominantly follows the Cawood and Howell Class IV pattern, resulting in a morphology that is fundamentally unsuitable for prosthetically driven implant placement [4].

Autogenous bone is widely considered the gold standard for three-dimensional ridge augmentation due to its osteoconductive, osteogenic, and osteoinductive properties. Its significant regenerative potential, along with the availability of both intraoral and extraoral donor sites, makes it particularly effective for reconstructing large alveolar defects prior to implant placement [32]. However, traditional block graft techniques require the harvesting of thicker blocks to address high post-operative resorption rates, which can range from 10% to 50%.

In this case series, the split bone block (SBB) technique was employed as a preliminary method for addressing deficient ridges prior to implantation. Clinically, this technique exhibited favorable handling characteristics and effectively maintained ridge contours during the healing period. By stabilizing the cortical bone plate with osteosynthesis screws and using particulate autogenous bone to fill the gap between the two bone plates or between one plate and the native bone, the graft surface area was likely increased. This enhancement may have facilitated early revascularization and graft integration. Previous literature supports the dimensional stability of this approach; Mertens et al. [33] compared full block grafting with split bone block grafting, reporting that the SBB technique resulted in superior horizontal and vertical augmentation outcomes in both anterior and posterior regions. Preliminary observations from the current study seem to align with these findings; however, the limited sample size and short-term follow-up warrant cautious interpretation of the results.

Radiographic evaluation using cone-beam computed tomography (CBCT) demonstrated a horizontal ridge gain. The mean preoperative bone width increased from 3.27 ± 0.80 mm to a post-operative mean of 5.88 ± 0.82 mm at 4 months, indicating a relative increase of 79.82%. Within the confines of this cohort, the difference in pre- and post-operative ridge width measurements suggests that the technique is clinically feasible.

These results support the findings of Ali et al. [34], who assessed horizontal ridge augmentation using autogenous block grafts and reported a mean increase in bone width of approximately 3.9 mm after six months, resulting in a statistically significant relative increase of 80.78%. While the relative percentage gain in the current study is consistent with that reported by Ali et al. [34], the greater absolute millimeter gain in their cohort may be due to variations in baseline ridge dimensions or initial graft volumes. Furthermore, a systematic review by Sanchez-Sanchez et al. [35] noted that horizontal bone gain achieved through the split bone block technique ranged from 3.93 ± 0.9 mm to 5.0 ± 0.8 mm, reinforcing the clinical feasibility observed in our study. Long-term studies suggest that the SBB approach provides excellent dimensional stability, with an 8.3% resorption rate documented over a 10-year period [17, 33, 36].

Achieving optimal implant stability is crucial for predictable osseointegration and long-term clinical success. While implants with lower Resonance Frequency Analysis (RFA) values can still achieve clinical outcomes, greater primary stability suggests better bone-to-implant contact (BIC), which reduces interfacial micromotion during the early healing phase. Vallecillo-Rivas et al. [37] documented a mean Implant Stability Quotient (ISQ) of 66 for implants placed in regenerated bone after a 12-week healing period. In contrast, the current study reported a higher mean ISQ of 73, indicating strong primary stability. Although these findings are preliminary, existing literature shows a positive correlation between ISQ values, bone mineral density, and overall bone quality [38]. Additionally, Herekar et al. [39] introduced a diagnostic index for bone classification, noting that an ISQ of 70.55 is typical of Type D2 (dense trabecular) bone morphology, which suggests favorable bone formation at our regenerated sites.

To establish essential baseline benchmarks for this descriptive cohort, early implant health and survival metrics were classified using the four-tier Misch et al. Pisa Consensus Scale [29]. Following the early evaluation window, eight implants (72.7%) achieved complete clinical success (Group I) by remaining completely asymptomatic and immobile, while two implants (18.2%) maintained satisfactory survival (Group II). One early implant failure (9.1%) occurred due to a localized loss of mechanical stability (Group IV), requiring removal. Postoperative complications in this study were mild and clinically manageable. Swelling was the most frequent complication, occurring in 5 of 11 patients, and it resolved spontaneously within 7 days. This swelling was strictly confined to a single anatomical area, reflecting the localized nature of the single-incision design where the donor and recipient sites coincide. Flap dehiscence and hematoma each affected two patients (18.18%).

The observed mucosal dehiscence ranged from minor screw exposure to graft exposure. According to the classification by Fontana et al. [40], the case of screw exposure involved less than 3 mm of tissue separation, qualifying it as Class I. Although this site did not achieve complete re-epithelialization over the screw, it still positively influenced the overall healing of the underlying grafted bone. In contrast, the graft exposure case exhibited tissue separation exceeding 3 mm without purulent exudate, qualifying as Fontana Class II, and achieved complete re-epithelialization within 14 days. This occurrence of dehiscence may be associated with cumulative surgical trauma from conducting both harvesting and grafting at the same site; however, this conclusion remains speculative due to the preliminary nature of this case series.

Maintaining primary closure is a crucial factor for successful guided bone regeneration. Historical evidence suggests that bone formation can be approximately six times greater in areas that achieve uninterrupted soft tissue healing compared to those compromised by early graft exposure [41]. In a landmark systematic review, Starch-Jensen et al. [42] reported a high prevalence of postoperative complications associated with alternative approaches, noting soft tissue dehiscence and subsequent exposure of allogeneic bone blocks in 27% to 74% of cases. In contrast, the single-incision split bone block technique employed in this series limited soft tissue dehiscence to 18.18% (2 out of 11 patients). The soft tissue healing parameters observed in our cohort are comparable to the findings of Velazquez et al. [43], who noted a 15% incidence of mucosal dehiscence (3 out of 16) following suture removal. In a retrospective study by Bayram et al. [44] evaluating complications during block grafting from the mandibular ramus, the incidence of dehiscence was 22.86%, which is slightly higher than the 18.18% noted in our study. However, Bayram et al. [44] reported a hematoma rate of 7.14% and a total graft loss rate of 14.29%. While our hematoma rate was higher, our approach avoided total graft loss entirely. Given the preliminary nature of these findings, the existing literature is integrated to contextualize our data and facilitate a deeper interpretation of the outcomes.

A clinical advantage of the single-site protocol is the minimization of donor-site morbidity, which correlates with improved postoperative recovery profiles and enhanced patient well-being. A systematic review of the complications encountered after conventional autogenous block harvesting from the symphysis evaluated common morbidity vectors. The most frequent complication encountered was temporary sensory disturbance to the teeth (33.87%) and skin (18.57%), followed by permanent neurosensory deficits (12.02%), bruising (7.1%), skin blemishes (5.46%), wound dehiscence (1.63%), and pulp necrosis (1.09%). Notably, none of the patients in our study experienced temporary or permanent neurosensory disturbances. This complete absence of neurosensory deficits stands in contrast to conventional donor-site literature, which reports paresthesia rates ranging from 1.8% to 33.8% [45, 46]. This clinical improvement may be attributed to avoiding the larger trephines and aggressive surgical burs often required for traditional block harvesting.

To better contextualize these findings within contemporary reconstructive literature, our single-site outcomes can be compared with a prospective comparative study by Fanai et al. [47]. Fanai et al. documented that traditional multi-site surgical protocols involving secondary harvesting from the mandibular ramus were frequently associated with pronounced donor-site complications, including localized swelling, restricted mouth opening, and transient paresthesia. By executing both bone harvesting and recipient augmentation within the boundaries of a single surgical window, the current case series minimized cumulative tissue trauma, resulting in a favorable single-zone complication profile and a complete absence of temporary or permanent neurosensory deficits.

The true scientific and clinical feasibility of the proposed protocol lies not in altering the established biological mechanisms of autogenous bone healing, but in the strategic spatial optimization of the surgical field. Traditional autogenous block or split bone grafting protocols are inherently limited by their reliance on secondary, distant donor sites, such as the mandibular ramus or a distinct symphysis zone. This multi-site approach essentially subjects the patient to two independent surgical wounds, which multiplies cumulative tissue trauma, increases immediate postoperative inflammation, and exposes the patient to distinct regional risks like permanent neurosensory damage, tooth vitality loss, and prolonged donor-site pain. By consolidating the donor harvest and the recipient augmentation within a singular, localized anatomical window, this single-incision technique limits surgical trauma strictly to the defect zone. We suggest that the single-zone confinement may minimize the overall biological burden on the patient, accelerate early functional recovery, and preserve regional neurosensory integrity. Consequently, this protocol addresses a critical historical barrier in autogenous grafting by directly enhancing patient-centered outcomes and postoperative quality of life, demonstrating that the clinical outcomes may be achieved through conservative surgical refinement rather than changing the biomaterial itself. This aligns with the findings of Chappuis et al. [48], who demonstrated that secondary donor site operations are the primary drivers of deteriorated patient perception and physical function during early healing phases.

A similar positive trend was observed regarding patient-reported discomfort. Velazquez et al. [43] reported that 70% of patients subjected to autogenous bone harvesting from the mandibular ramus experienced moderate postoperative pain. In their protocol, pain severity was indexed to analgesic consumption, where consuming fewer than 4 paracetamol tablets indicated mild pain, 4 to 8 tablets indicated moderate pain, and more than 8 tablets indicated severe pain. They also noted that only 36% of patients experienced moderate pain when allogeneic blocks were used, thus avoiding a secondary surgery. The dual-site surgical approach utilized by Velazquez et al. for harvesting from the ramus and grafting elsewhere likely accounts for this high morbidity.

In our study, confining the surgical intervention to a single zone resulted in manageable patient-reported discomfort. The mean Visual Analog Scale (VAS) pain score tracked downward from 5.73 ± 1.56 at one week to 2.18 ± 2.40 by the second postoperative week, representing a 61.95% reduction in pain levels. Most patients characterized the pain as mild to moderate and easily manageable with standard analgesics. While these primary results are encouraging, the lack of an internal control group and the small sample size restrict any definitive comparative conclusions. Future validation in larger, randomized controlled clinical trials is required to confirm these preliminary outcomes.

### Strengths

Split bone block is a well-established technique. Autogenous bone is the gold standard for augmentation. The hassle of two surgeries and donor site morbidity has been a drawback to this technique.

- All the results align with literature data and previous clinical trials with fewer complications.
- The patients were compliant, and the follow-up policy was strict.

### Weaknesses and limitations

While our single-site modification offers the biological benefits of autogenous bone within a localized surgical window, several inherent limitations must be acknowledged:

- Lack of a Control Group: The absence of a randomized control or direct comparative group prevents definitive conclusions regarding superior healing or reduced morbidity.
- Small Sample Size: The cohort is limited to 11 patients, which restricts the external validity and generalizability of our preliminary findings.
- Short Follow-Up Window: The ~ 7-month follow-up period is insufficient to evaluate long-term dimensional graft stability or peri-implant bone behavior under prolonged functional loading.
- Lack of Blinding: The nature of this clinical case series and the absence of blinded outcome assessors may introduce investigator bias during clinical and radiographic measurements.
- Anatomical Constraints: The technical feasibility relies entirely on having a viable donor block site immediately adjacent to the horizontal defect, limiting its application to specific clinical scenarios.
- Absence of Volumetric Analysis: Radiographic tracking was limited to localized linear width measurements (mm). The lack of consecutive, volumetrically standardized CBCT datasets prevented 3D volume segmentation and graft voxel analysis.
- Defect Heterogeneity and number of bone plates used: Baseline defect dimensions and morphology were not strictly standardized between subjects, requiring clinical adaptation between utilizing one versus two cortical bone plates. This adaptive plating introduces a degree of clinical heterogeneity and confounding variables, which limits the uniform isolation of outcome factors within this small sample.

### Directions for future research

We encourage future investigations on:

- Clinical trials with larger sample sizes and longer follow-up periods.
- The effect of defect size on the clinical outcomes.
- The morbidity of different sites for the same operation.
- The resorption rate and the grafted bone stability.
- The application of standardized 3D volumetric segmentation software in future trials to accurately quantify three-dimensional graft volume changes and resorption rates.

### Cost

This study provides a more economical option for ridge augmentation with fewer complications and comparable results to both xenogeneic and allogenic techniques for augmentation.

## Conclusions

This study’s results suggest that the split bone block technique from the same site as both donor and recipient seemed to achieve desired clinical outcomes, including manageable postoperative complications, effective pain reduction, bone width enhancement, and improved implant stability over time. These findings contribute valuable insights into optimizing surgical techniques and patient care protocols in dental implantology. However, this observation needs further replication in larger randomized controlled clinical trials to ensure effectiveness and compare results.

## Data Availability

All data supporting the findings of this study are available within the paper and its Supplementary Information.

## Abbreviations

GBR: Guided bone regeneration
SBB: Split Bone Block
MM: Millimetres
SD: Standard Deviation
PI: Plaque Index
PPD: Probing Pocket Depth
BOP: Bleeding On Probing
CBCT: Cone Beam Computed Tomography
ISQ: Implant Stability Quotient
RFA: Resonance Frequency Analysis
DICOM: Digital Imaging and Communication in Medicine
MPR: Multiplanar Reformation
VAS: Visual Analogue Scale
BIC: Bone to Implant Contact

## References

[1] Buser D, Janner SF, Wittneben JG, Brägger U, Ramseier CA, Salvi GE. 10-year survival and success rates of 511 titanium implants with a sandblasted and acid-etched surface: a retrospective study in 303 partially edentulous patients. Clin Implant Dent Relat Res. 2012;14:839–51. 22897683 10.1111/j.1708-8208.2012.00456.x

[2] Lin HK, Pan YH, Salamanca E, Lin YT, Chang WJ. Prevention of bone resorption by HA/β-TCP + collagen composite after tooth extraction: a case series. Int J Environ Res Public Health. 2019;16:4616. 31766327 10.3390/ijerph16234616 PMC6926561

[3] Couso-Queiruga E, Stuhr S, Tattan M, Chambrone L, Avila-Ortiz G. Post-extraction dimensional changes: a systematic review and meta-analysis. J Clin Periodontol. 2021;48:126–44. 33067890 10.1111/jcpe.13390

[4] Alshenaiber R, Cowan C, Barclay C, Silikas N. Analysis of residual ridge morphology in a group of edentulous patients seeking NHS dental implant provision-a retrospective observational lateral cephalometric study. Diagnostics (Basel). 2021;11:2348. 34943585 10.3390/diagnostics11122348 PMC8700105

[5] Zmysłowska E, Ledzion S, Jedrzejewski K. Factors affecting mandibular residual ridge resorption in edentulous patients: a preliminary report. Folia Morphol (Warsz). 2007;66:346–52. 18058759

[6] Cawood JI, Howell RA. A classification of the edentulous jaws. Int J Oral Maxillofac Surg. 1988;17:232–6. 3139793 10.1016/s0901-5027(88)80047-x

[7] Stimmelmayr M, Güth JF, Schlee M, Göhring TN, Beuer F. Use of a modified shell technique for three-dimensional bone grafting: description of a technique. Aust Dent J. 2012;57:93–7. 22369565 10.1111/j.1834-7819.2011.01646.x

[8] Lim G, Lin GH, Monje A, Chan HL, Wang HL. Wound healing complications following guided bone regeneration for ridge augmentation: a systematic review and meta-analysis. Int J Oral Maxillofac Implants. 2018;33:41–50. 28938030 10.11607/jomi.5581

[9] Smeets R, Matthies L, Windisch P, Gosau M, Jung R, Brodala N, Stefanini M, Kleinheinz J, Payer M, Henningsen A, Al-Nawas B, Knipfer C. Horizontal augmentation techniques in the mandible: a systematic review. Int J Implant Dent. 2022;8:23. 35532820 10.1186/s40729-022-00421-7 PMC9086020

[10] Silva ER, Ferraz EP, Neto EC, Chaushu G, Chaushu L, Xavier SP. Volumetric stability of fresh frozen bone blocks in atrophic posterior mandible augmentation. J Oral Implantol. 2017;43:25–32. 27753539 10.1563/aaid-joi-D-16-00095

[11] Sakkas A, Wilde F, Heufelder M, Winter K, Schramm A. Autogenous bone grafts in oral implantology-is it still a gold standard? A consecutive review of 279 patients with 456 clinical procedures. Int J Implant Dent. 2017;3:23. 28573552 10.1186/s40729-017-0084-4 PMC5453915

[12] Petrie A, Sabin C. Medical statistics at a glance. 3rd ed. Chichester, West Sussex: Wiley; 2009.

[13] Meloni SM, Jovanovic SA, Urban I, Baldoni E, Pisano M, Tallarico M. Horizontal ridge augmentation using GBR with a native collagen membrane and 1:1 ratio of particulate xenograft and autologous bone: A 3-year after final loading prospective clinical study. Clin Implant Dent Relat Res. 2019;21:669–77. 31286654 10.1111/cid.12808

[14] Tarun Kumar AB, Triveni MG, Priyadharshini V, Mehta DS. Staged ridge split procedure in the management of horizontal ridge deficiency utilizing piezosurgery. J Maxillofac Oral Surg. 2016;15:542–6. 27833350 10.1007/s12663-015-0790-5 PMC5083681

[15] Funaki K, Takahashi T, Yamuchi K. Horizontal alveolar ridge augmentation using distraction osteogenesis: comparison with a bone-splitting method in a dog model. Oral Surg Oral Med Oral Pathol Oral Radiol Endod. 2009;107:350–8. 19121955 10.1016/j.tripleo.2008.10.005

[16] Atef M, Osman AH, Hakam M. Autogenous interpositional block graft vs onlay graft for horizontal ridge augmentation in the mandible. Clin Implant Dent Relat Res. 2019;21:678–85. 31260166 10.1111/cid.12809

[17] Khoury F, Hanser T. Three-dimensional vertical alveolar ridge augmentation in the posterior maxilla: a 10-year clinical study. Int J Oral Maxillofac Implants. 2019;34:471–80. 30883623 10.11607/jomi.6869

[18] Agha RA, Mathew G, Rashid R, Kerwan A, Al-Jabir A, Sohrabi C, Franchi T, Nicola M, Agha M, Thoma A, Coppola A. Revised preferred reporting of case series in surgery (PROCESS) guideline: an update for the age of artificial intelligence. Premier Premier J Sci. 2025;10:100080. 10.70389/PJS.100080.

[19] Abouleish AE, Leib ML, Cohen NH. ASA provides examples to each ASA physical status class. ASA Monit. 2015;79:38–49.

[20] Koduganti RA, Harika RR, Rajaram TSL. Ridge augmentation is a prerequisite for successful implant placement: a literature review. Cureus. 2022;14:e20872. 35145779 10.7759/cureus.20872 PMC8805661

[21] Wang HL, Al-Shammari K. HVC ridge deficiency classification: a therapeutically oriented classification. Int J Periodontics Restor Dent. 2002;22:335–43. 12212680

[22] Hwang D, Wang HL. Medical contraindications to implant therapy: part I: absolute contraindications. Implant Dent. 2006;15:353–60. 17172952 10.1097/01.id.0000247855.75691.03

[23] Roshan J, Surej Kumar LK, Rahim SN, Adersh GA, Thuruthel MJ, Haris HA. Reconstruction of osseous defect with symphysis block graft for implant placement. J Oral Biol Craniofac Res. 2022;12:853–8. 36203858 10.1016/j.jobcr.2022.09.010 PMC9531279

[24] Botros MA, Gaber HK, Abbas EA, El-Mofty MS, Bissar MW. Split-block graft versus cortico-cancellous block graft for horizontal ridge augmentation: cone beam computed tomography and histomorphometric study. Braz dent sci. 2021;24(3):e2464. 10.14295/bds.2021.v24i3.2464.

[25] Silness J, Loe H. Periodontal disease in pregnancy. Ii. Correlation between oral hygiene and periodontal condtion. Acta odontol scand. 1964;22:121–35. 14158464 10.3109/00016356408993968

[26] Papapanou PN, Sanz M, Buduneli N, Dietrich T, Feres M, Fine DH, et al. Periodontitis: consensus report of workgroup 2 of the 2017 world workshop on the classification of periodontal and peri-implant diseases and conditions. J Periodontol. 2018;89(Suppl):S173–82. 29926951 10.1002/JPER.17-0721

[27] Hur Y, Bui MN, Griffin TJ, Ogata Y. Modified periosteal releasing incision for flap advancement: a practical technique for tensionless closure. Clin Adv Periodontics. 2015;5:229–34.

[28] Good M, Stiller C, Zauszniewski JA, Anderson GC, Stanton-Hicks M, Grass JA. Sensation and distress of pain scales: reliability, validity, and sensitivity. J Nurs Meas. 2001;9:219–38. 11881266

[29] Misch CE, Perel ML, Wang HL, Sammartino G, Galindo-Moreno P, Trisi P et al. Implant success, survival, and failure: the International Congress of Oral Implantologists (ICOI) Pisa Consensus Conference. Implant Dent. 2008;17:5–15. 10.1097/ID.0b013e3181676059 18332753

[30] Gupta G. Implant Stability Quotient (ISQ): A reliable guide for implant treatment. In: Gabric D, Vuletic M, editors. Current Concepts in Dental Implantology: From Science to Clinical Research. London: IntechOpen. 2022. 10.5772/intechopen.101359. Chapter 5.

[31] Osstell AB. Clinical guidelines. Sweden: Gothenburg. 2023 Available at: https://www.osstell.com/clinical-guidelines.

[32] Khoury F, Hanser T. Mandibular bone block harvesting from the retromolar region: a 10-year prospective clinical study. Int J Oral Maxillofac Implants. 2015;30:688–97. 26009921 10.11607/jomi.4117

[33] Mertens C, Büsch C, Goldenbaum K, Ristow O, Hoffmann J, Wang HL, Hoffmann KJ. Full block or split block?-Comparison of two different autogenous block grafting techniques for alveolar ridge reconstruction. Clin Implant Dent Relat Res. 2023;25:1149–63. 37584303 10.1111/cid.13263

[34] Kheir El Din ALIA. Horizontal alveolar ridge augmentation with autogenous block bone graft a cone beam computed tomography evaluation case series. Egypt Dent J. 2021;67:3193–202. 10.21608/edj.2021.82900.1694.

[35] Sánchez-Sánchez J, Pickert FN, Sánchez-Labrador L, Gf Tresguerres F, Martínez-González JM. Meniz-García C. Horizontal ridge augmentation: a comparison between khoury and urban technique. Biology (Basel). 2021;10:749. 34439981 10.3390/biology10080749 PMC8389589

[36] Pommer B, Zechner W, Watzek G, Palmer R. To graft or not to graft? Evidence-based guide to decision making in oral bone graft surgery. Bone grafting. 2012;2012:25.

[37] Vallecillo-Rivas M, Reyes-Botella C, Vallecillo C, Lisbona-González MJ, Vallecillo-Capilla M, Olmedo-Gaya MV. Comparison of implant stability between regenerated and non-regenerated bone. a prospective cohort study. J Clin Med. 2021;10:3220. 34362004 10.3390/jcm10153220 PMC8347999

[38] Ivanova V, Chenchev I, Zlatev S, Mijiritsky E. Correlation between primary, secondary stability, bone density, percentage of vital bone formation and implant size. Int J Environ Res Public Health. 2021;18:6994. 34208849 10.3390/ijerph18136994 PMC8297224

[39] Herekar M, Sethi M, Ahmad T, Fernandes AS, Patil V, Kulkarni H. A correlation between bone (B), insertion torque (IT), and implant stability (S): BITS score. J Prosthet Dent. 2014;112:805–10. 24726588 10.1016/j.prosdent.2014.02.011

[40] Fontana F, Maschera E, Rocchietta I, Simion M. Clinical classification of complications in guided bone regeneration procedures by means of a nonresorbable membrane. Int J Periodontics Restor Dent. 2011;31:265–73. 21556383

[41] Machtei EE. The effect of membrane exposure on the outcome of regenerative procedures in humans: a meta-analysis. J Periodontol. 2001;72:512–6. 11338304 10.1902/jop.2001.72.4.512

[42] Starch-Jensen T, Deluiz D, Tinoco EMB. Horizontal alveolar ridge augmentation with allogeneic bone block graft compared with autogenous bone block graft: a systematic review. J Oral Maxillofac Res. 2020;11:e1. 32377325 10.5037/jomr.2020.11101 PMC7191383

[43] Velázquez ÓI, Tresguerres FGF, Berrocal IL, Tresguerres IF, López-Pintor RM, Carballido J, López-Quiles J, Torres J. Split bone block technique: 4-month results of a randomised clinical trial comparing clinical and radiographic outcomes between autogenous and xenogeneic cortical plates. Int J Oral Implantol (Berl). 2021;14(1):41–52. Erratum in: Int J Oral Implantol (Berl). 2021;14:226. 34006070

[44] Bayram F, Göçmen G, Özkan Y. Evaluating risk factors and complications in mandibular ramus block grafting: a retrospective cohort study. Clin Oral Investig. 2024;28:226. 38514518 10.1007/s00784-024-05613-6 PMC10957589

[45] Andersen K, Nørholt SE, Knudsen J, Küseler A, Jensen J. Donor site morbidity after reconstruction of alveolar bone defects with mandibular symphyseal bone grafts in cleft patients–111 consecutive patients. Int J Oral Maxillofac Surg. 2014;43:428–32. 24183738 10.1016/j.ijom.2013.09.007

[46] Joshi A. An investigation of post-operative morbidity following chin graft surgery. Br Dent J. 2004;196:215–8. discussion 211. 15039731 10.1038/sj.bdj.4810987

[47] Fanai JV, Dhuvad J, Anchlia S, Bhatt U, Trivedi B, Jogani H. Quantitative evaluation of zygomatic buttress, ramal, and symphysis onlay grafts in ridge augmentation prior to implant placement. J Maxillofac Oral Surg. 2025;24:1575–84. 41306213 10.1007/s12663-024-02192-7 PMC12644274

[48] Chappuis V, Rahman O, Buser R, Bornstein MM, Belser UC, Buser D. Clinical relevance of donor site morbidity and patient’s perception after autogenous bone harvesting from the mandibular ramus: a prospective study. Clin Oral Implants Res. 2010;21(11):1216–25.

